# PS1/gamma-secretase acts as rogue chaperone of glutamate transporter EAAT2/GLT-1 in Alzheimer’s disease

**DOI:** 10.21203/rs.3.rs-3495211/v1

**Published:** 2023-11-07

**Authors:** Florian Perrin, Lauren C. Anderson, Shane P.C. Mitchell, Priyanka Sinha, Yuliia Turchyna, Masato Maesako, Mei C.Q. Houser, Can Zhang, Steven L. Wagner, Rudolph E. Tanzi, Oksana Berezovska

**Affiliations:** Massachusetts General Hospital; Massachusetts General Hospital; Massachusetts General Hospital; Massachusetts General Hospital; Massachusetts General Hospital; Massachusetts General Hospital; Massachusetts General Hospital; Massachusetts General Hospital; VA San Diego Healthcare System; Massachusetts General Hospital; Massachusetts General Hospital

**Keywords:** Alzheimer’s disease, Presenilin 1, EAAT2, GLT-1, Glutamate transport, Hyperactivity, Gamma-secretase, Chaperone

## Abstract

The recently discovered interaction between presenilin 1 (PS1), a catalytic subunit of γ-secretase responsible for the generation of amyloid-β(Aβ) peptides, and GLT-1, the major glutamate transporter in the brain (EAAT2 in the human) may provide a mechanistic link between two important pathological aspects of Alzheimer’s disease (AD): abnormal Aβoccurrence and neuronal network hyperactivity. In the current study, we employed a FRET-based approach, fluorescence lifetime imaging microscopy (FLIM), to characterize the PS1/GLT-1 interaction in its native environment in the brain tissue of sporadic AD (sAD) patients. There was significantly less interaction between PS1 and GLT-1 in sAD brains, compared to tissue from patients with frontotemporal lobar degeneration (FTLD), or non-demented age-matched controls. Since PS1 has been shown to adopt pathogenic “closed” conformation in sAD but not in FTLD, we assessed the impact of changes in PS1 conformation on the interaction. Familial AD (fAD) PS1 mutations which induce a “closed” PS1 conformation similar to that in sAD brain and gamma-secretase modulators (GSMs) which induce a “relaxed” conformation, reduced and increased the interaction, respectively. This indicates that PS1 conformation seems to have a direct effect on the interaction with GLT-1. Furthermore, using biotinylation/streptavidin pull-down, western blotting, and cycloheximide chase assays, we determined that the presence of PS1 increased GLT-1 cell surface expression and GLT-1 homomultimer formation, but did not impact GLT-1 protein stability. Together, the current findings suggest that the newly described PS1/GLT-1 interaction endows PS1 with chaperone activity, modulating GLT-1 transport to the cell surface and stabilizing the dimeric-trimeric states of the protein. The diminished PS1/GLT-1 interaction suggests that these functions of the interaction may not work properly in AD.

## Introduction

One of the hallmarks of the neurodegenerative disease Alzheimer’s disease (AD) is the formation of extracellular amyloid-beta (Aβ) plaques. Presenilin 1 (PS1), a catalytic subunit of γ-secretase, has been established as a critical player in the development of AD pathology in part because PS1 is responsible for the cut that determines the length of the amyloid β (Aβ) peptides [14, 70]. Familial AD (fAD) mutations in the PS1 protein result in pathogenic conformational changes that correlate with an increased Aβ 42/40 ratio and increased plaque formation [5, 8, 64]. In the search for potential interactors of PS1, we previously identified a novel interaction between the PS1 and glutamate transporter 1 (GLT-1 in rodents, excitatory amino acid transporter 2, EAAT2, in humans), the major glutamate transporter in the brain [77].

Hyperactivity, epileptic seizures, and impaired glutamate transport occur early in AD pathology prior to most other known changes, including cognitive decline [7, 19, 40, 54, 66]. In fact, alterations in glutamate transporter expression precedes amyloid plaque formation in AD mouse models [9, 23, 48] and glutamate dysregulation occurs prior to and positively correlates with cognitive decline in humans [7, 15, 60]. EAAT2/GLT-1 is responsible for a majority of glutamate uptake from synapses [56, 57] and has been implicated both in mouse models and in humans as having a large role in glutamate dysfunction [22, 44, 46, 49, 73, 79]. Notably, upregulation of GLT-1 in an AD mouse model alleviated cognitive deficits [16], and higher GLT-1 expression was observed in brains from individuals with AD pathology but not dementia compared to those of individuals with AD pathology and dementia [30]. However, the physiological modulators of the aberrant glutamate uptake in AD, and what might be causing GLT-1 to become dysregulated, are not yet well understood.

We have recently reported that GLT-1 directly binds and interacts with PS1/γ-secretase [77] and have hypothesized that the PS1 and GLT-1 interaction is a functional one that may become dysregulated in AD. Previous reports have linked PS1/amyloid pathology to glutamate activity: AD patients, particularly those with mutations in PS1, have a higher incident of epileptiform activity [12, 47]. Moreover, patients with childhood epilepsy, when examined 50 years later, exhibited an increase in amyloid load, a risk factor for developing AD later in life [27]. PS1 deficient neurons had a significant decrease in glutamate uptake [74] and GLT-1 deficiency in an amyloid-β precursor protein and PS1 fAD mouse model increased memory deficits as well as the ratio of Aβ_42_/Aβ_40_ [45]. Treatment with amyloid-beta peptide Aβ_1–40_ decreased GLT-1 uptake capacity in primary rat astrocyte cultures [41] and treatment with Aβ_1–42_ reduced GLT-1 cell surface expression in mouse brain hippocampal slices [58]. Therefore, the interaction between PS1 and GLT-1 that we identified [77] may be the link between two major pathological aspects of AD and could provide a potential novel therapeutic target for AD.

The current studies investigated the molecular link between PS1 and GLT-1 by examining its interaction in sporadic AD (sAD), FTLD, and Control human brains. Additionally, we assessed the impact of changes in PS1 conformation due to fAD PS1 mutations and γ-secretase modulators (GSMs) on the interaction, and explored PS1 effect on GLT-1 cell surface expression, multimerization, and stability. We found increased GLT-1 aggregation and disrupted PS1/GLT-1 interaction in sAD human brain, and revealed that PS1 potentiates GLT-1 cell surface expression and multimerization. We show the interaction is affected *in vitro* by PS1 conformational changes in a way that may affect GLT-1’s normal cellular functioning in the brain.

## Materials and Methods

### Human Brain Tissue

Human brains from neuropathologically verified AD or Frontotemporal lobar degeneration (FTLD) cases were obtained from the brain bank of the Alzheimer’s Disease Research Center (ADRC) at Massachusetts General Hospital. Control cases were non-demented individuals who did not meet pathological diagnostic criteria of AD, FTLD or other neurodegenerative diseases (Supplementary Tables 1 and 2). All the subjects or their next of kin gave informed consent for the brain donation. Hippocampal and frontal cortex sections were used for immunohistochemistry; frontal cortex samples were used for Western blotting/immunoprecipitation. This article does not contain any studies with human participants or animals performed by any of the authors.

### Cell cultures and transfection

Chinese hamster ovary (CHO) cells were maintained in OPTI MEM medium supplemented with 5% FBS in a 37°C CO_2_ incubator. Human embryonic kidney (HEK) cells in which PS1 and PS2 are knocked down (HEK PS DKO) were kindly provided by Dr. Dennis Selkoe, BHW, Boston, MA, and were maintained in DMEM supplemented with 5% FBS, 1% GlutaMax and 1% Pen/Strep mix (Life Technologies, Carlsbad, CA) in a 37°C CO_2_ incubator. Lipofectamine 3000 (Life Technologies, Carlsbad, CA) was used for transient transfection according to the manufacturer’s instructions.

HEK PS DKO and HEK wt were transduced with lentivirus expressing GLT-1 GFP and selected with 10 μg/mL of puromycin to generate HEK PS DKO GLT-1 and HEK wt GLT-1 stable cell lines, respectively.

### Plasmid constructs

Plasmids encoding PS1wt and PS1 fAD were cloned into pcDNA3.1 vector (Addgene). The GLT-1 encoding sequence was subcloned into pcDNA^™^6 V5 Myc (ThermoScientific). The GFP sequence cloned in pcDNA3.1 was used as negative control for all experiments, except for flow cytometry where pcDNA3.1 empty vector was used and referred to as Mock. Lentivirus production used packaging plasmids psPAX2 and pMD2.G (Addgene), and a third vector pLenti-SLC1A2-C-mGFP-P2A-Puro (Origene).

### Drug Treatments

In the experiments using γ-secretase modulators (GSMs), cells were treated with 20 nM GSM15606 [52], 3 μM GSM36 [68] or dimethyl sulfoxide (DMSO) vehicle control for 16 hours. GLT-1 selective inhibitors, DHK (dihydrokainate) and WAY213613 were obtained from Tocris.

### Immunocytochemistry (ICC) and Immunohistochemistry (IHC)

PFA-fixed brain tissue was sectioned into 50μm-thick sections on a Leica freezing microtome (Leica SM 2000R, Bannockburn, IL) and used for IHC immunostaining. For ICC, *in vitro* cultured cells were washed twice with Dulbecco’s Phosphate Buffered Saline without Calcium Chloride or Magnesium Chloride (PBS; ThermoScientific, Waltham, MA) and fixed by 15-minute incubation with 4% PFA. Following the fixation, free-floating brain sections or cells were permeabilized using 0.1% TX-100 in a 1.5% normal donkey serum (NDS; Jackson ImmunoResearch labs, West Grove, PA) blocking solution for 1 hour. After three 5 min washes in PBS, samples were incubated overnight with respective primary antibodies in 1.5% NDS. Excess primary antibodies were washed off with three 5 min washes in PBS and the corresponding Alexa Fluor 488- or Cy3-conjugated secondary antibodies (1:500) were applied for 1 hour at room temperature. Cells and tissue were then washed three additional times in PBS. Cells were coverslipped with VectaShield mounting medium (Vector Laboratories, Inc., Burlingame, CA) and brain sections were mounted onto microscope slides and coverslipped with VectaShield.

### Antibodies

The following primary antibodies were used: guinea pig anti-GLT-1 (AB1783, EMD Millipore, Temecula, CA) and rabbit anti-GLT-1 (ab41621, Abcam, Cambridge, MA), both targeting C-term region; rabbit nGLT-1 targeting N-term [11] (kindly provided by our collaborator, Dr. Rosenberg, BCH, Boston), rabbit anti-GLT-1 (NBP120136, Novusbio) undisclosed epitope; rabbit anti-PS1 raised against a.a.263–378 of PS1 (S182, Sigma-Aldrich, St. Louis, MO); mouse anti-PS1 raised against N-term (Biolegends, 823401), mouse anti-β-actin (A2228, Sigma-Aldrich, St. Louis, MO); rabbit anti-PS2 (mAb 9979, Cell Signaling Technology, Danvers, MA); EGFR (ab52894, Abcam, MA); normal rabbit IgG (2728S, Cell Signaling Technology, Danvers, MA). Alexa Fluor 488 (ThermoScientific, Waltham, MA) and Cy3-conjugated secondary antibodies (Jackson ImmunoResearch, West Grove, PA) were applied for the microscopy imaging and IRDye680/800-(Li-COR, Lincoln, NE) labelled ones were used for western blotting.

### Fluorescence lifetime imaging microscopy (FLIM)

FLIM assay to monitor relative proximity between fluorescently labeled molecules in intact cells/tissue was conducted as described previously [69, 77]. We measured fluorescence lifetime rather than intensity to determine the Forster resonance energy transfer (FRET) efficiency. Fluorescence lifetime is an intrinsic biophysical property of the fluorophore and, unlike fluorescence intensity, does not depend on the concentration or stoichiometry of the donor and acceptor fluorophores [4, 33]. Briefly, cells were immunostained with anti-GLT-1 CT (1:250) and anti-PS1 (loop) (1:250) antibodies. Corresponding secondary antibodies conjugated with Alexa Fluor 488 (AF488) and Cy3 fluorophores were used as the donor and acceptor, respectively. A sample in which the acceptor antibody was omitted was used as a negative control to record the baseline lifetime (*t*1) of the donor fluorophore. A femtosecond-pulsed Chameleon Ti:Sapphire laser (Coherent Inc., Santa Clara, CA) at 850 nm was used for two-photon fluorescence excitation. AF488 fluorescence was acquired using an emission filter centered at 515/30 nm. The donor fluorophore lifetimes were measured with a high-speed photomultiplier tube (MCP R3809; Hamamatsu, Bridgewater, NJ) and a fast time-correlated single-photon counting acquisition board (SPC-830; Becker & Hickl, Berlin, Germany). The data were analyzed using SPCImage software (Becker and Hickl, Berlin, Germany). The AF488 lifetimes were calculated by fitting raw data to the single-exponential (AF488 negative control) or two-exponential (AF488- and Cy3-double immunostained sample) fluorescence decay curves. *t*_*2*_ Lifetime values that are shorter than *t*_*1*_ indicate the presence of the FRET, i.e., less than 5–10 nm distance between the donor and acceptor fluorophores. FRET efficiency was calculated by subtracting the measured lifetime (*t*2) from the baseline lifetime *(t*1), divided by t1, expressed as a percentage (i.e. [(t1−t2)/t1]*100).

For human brain tissue, we used three-component analysis of the fluorescence lifetimes to exclude interference of the non-specific signal from autofluorescence/lipofuscin as previously described [69]. Briefly, the baseline lifetimes of AF488 and autofluorescence were calculated by fitting raw data to two-exponencial decay curves (negative control) to determine the value of t1 (autofluorescence, short lifetime ~ 400 psec) and t2 (A488, long lifetime comparable to that in cells *in vitro*). Then the brain samples immunostained with AF488 and Cy3-labeled antibodies against GLT-1 (1:250) and PS1 (1:250) were analyzed using three-component analysis. The AF488 donor fluorophore lifetime shortening due to FRET (t3) was then determined by fixing and removing from the analysis t1 (autofluorescence) and t2 (baseline AF488) [69].

### Biotinylation of Cell Surface Proteins

To isolate cell surface proteins, HEK PS DKO cells were transiently transfected with pcDNA3.1-GFP (pc) alone, or co-transfected with GLT-1 and pcDNA3.1-GFP or GLT-1 and PS1. All transfections are at 1:1 ratio. The cells were labelled with biotin using an ABCAM kit according to the manufacturer’s instructions (ab206998). Pulled down proteins were analyzed by Western blot.

### Immunoprecipitation (IP), Western blotting (WB), and Native Blue PAGE

Flash frozen human brain tissue was lysed in 1% 3-[(3-cholamidopropyl) dimethylammonio]-2-hydroxy-1-propanesulfonate (CHAPSO) in buffer (50 mM (4-(2-hydroxyethyl)-1-piperazineethansefulfonic acid [HEPES], 100 mM NaCl, pH 7.4) with Halt protease and phosphatase inhibitor cocktail (Fisher Scientific, Pittsburg, PA, USA). To homogenize the tissue, it was agitated with a pipette 50 times, agitated with a 27guage needle 10 times, rotated at 4°C for 1 hour and then centrifuged for 15 min at 14,000g at 4°C. Total protein in the final supernatants was measured using ThermoScientific Pierce BCA Protein Assay (ThermoScientific, Waltham, MA) following the supplier’s protocol. Lysis of freshly dissected mouse brain tissue and harvested cells followed the same protocol. Western blotting was performed on the same day as cell/tissue lysis.

For the IP procedure, 50 μl of Protein G Dynabeads (ThermoScientific, Waltham, MA) was incubated with 5 μg of the respective antibody or normal IgG, as a negative control, for 10 min at room temperature. The beads were then collected using a magnet and washed in 0.02%Tween 20 (Sigma-Aldrich, St. Louis, MO) in PBS using a magnetic tube rack. Aliquots of the supernatant containing equal amount of protein (500ug in 400ul) were then added to the beads and incubated overnight at 4°C with end-over-end rotation. The beads coupled with the complexes were collected, washed three times with 0.02%Tween and transferred to a new tube. The elution was performed by boiling the samples for 5 minutes in 30 μl Elution buffer (50mM Glycine, LDS, and DTT-containing reducing agent buffer [ThermoScientific, Waltham, MA]).

To resolve the proteins from the co-immunoprecipitation, biotinylation and cycloheximide preparations, the samples were loaded on 4–12% Bis-Tris NuPage polyacrylamide gels (ThermoScientific, Waltham, MA) and transferred to nitrocellulose membranes (GE Healthcare Lifesciences, Pittsburgh, PA) using BioRad system. The detection was performed by immunoblotting with specific primary and corresponding IRdye680/800-conjugated secondary antibodies (1:5000) and the bands were visualized using Odyssey Infrared Imaging System (Li-COR, Lincoln, NE). The quantitative analysis of the respective bands’ optical density was performed using ImageStudio Lite Ver 5.2 software.

To stabilize potential GLT-1 dimers/trimers we employed disuccinimidyl suberate (DSS) crosslinking agent. After GLT-1/PS1 transfection, HEK PS DKO cells were washed with Ca^2+^ and Mg^2+^ free PBS (PBS−/−). Crosslinking was performed in PBS−/− using DSS dissolved in DMSO at final concentration of 50 μM for 30 min at room temperature and then quenched with 20 mM glycine. Cells were lysed in 1% NP-40 + 0.1% Triton-X buffer. Fresh lysates were analyzed by western blotting with GLT-1 antibody.

For Blue Native PAGE, the cells were lysed in native blue buffer, digitonin 5% (Invitrogen) and 100X protease inhibitor (Pierce). For human brain tissue, approximately 20 mg of frozen cortical brain tissue was homogenized in 500 μl of 1× NativePAGE sample buffer with digitonin 5% (Invitrogen). After centrifugation (25,000g, 1 h at 4°C) supernatants were collected (30 μg of total protein) and 5% of the sample buffer was added. Protein lysates were separated using the Novex^®^ NativePAGE^™^ Bis-Tris gel system (Invitrogen). The electrophoresis and transfer were performed as per manufacturer’s instructions. Membranes were incubated overnight at 4°C with anti-GLT-1 primary antibody (ABCAM) at dilutions 1:1,000 and anti-rabbit HRP at 1:5,000. Quantification of the bands was performed with ImageJ using area under the curve method.

### Flow cytometry

Flow cytometry analysis was used to characterize cell surface expression of GLT-1. CHO cells were transiently transfected with pcDNA3.1 alone (pc) or co-transfected at 1:1 ratio with GLT-1 and pc or GLT-1 and PS1 to evaluate PS1 effect on cell surface GLT-1. At least 300,000 cells were detached with EDTA, fixed with 2% PFA and incubated with GLT-1 antibody (Novusbio) for 1h at 4°C with dilution at 1:100 (in 100 μL of PBS with 5% BSA and 2 μM EDTA) followed by PE-conjugated monoclonal antibody (ABCAM) at 1:500 dilution. “Mock” corresponds to CHO cells without GLT-1 transfected only with pcDNA3.1 empty vector and probed with rabbit IgG Isotype control and PE conjugated antibody.

Labeled cells were analyzed by flow cytometry on MACS Quant VYB flow cytometer (Myltenyi Biotec) using FlowJo V10 software. Alternatively, HEK PS DKO and HEK wt cells stably expressing GLT-1 GFP were used without fixation and permeabilization. GFP-GLT-1 signal was measured in the same number of HEK PS DKO GLT-1 GFP and HEK wt GLT-1 GFP cells expressing either PS1 or pcDNA3.1 (pc). “Mock” corresponds to HEK PS DKO cells without GLT-1 GFP and transfected only with pcDNA3.1 empty vector.

### Cycloheximide (CHX) assay

Relative protein stability was estimated using a CHX assay. CHO cells co-transfected at 1:1 ratio with GLT-1 and PS1, empty vector, or eGFP, were treated with 20 μg/ml CHX (Sigma-Aldrich) diluted in growth medium. The cells were collected in 0, 4, 8, 24, 32, 48 hr time points following the addition of CHX and lysed in 1% CHAPSO buffer with protease and phosphatase inhibitor cocktail. The extracted proteins were analyzed by western blotting. The intensity of each band was quantified using ImageStudio Lite Ver. 5.2 and normalized to the level of protein at the “0” time point. The data were plotted on a graph, fitted with a one-phase decay curve, and analyzed with a Two-way ANOVA followed by Dunnett’s multiple comparisons test, using GraphPad Prism 10 software.

### Glutamate uptake assay

Previously published procedures were followed for measuring the uptake of glutamate into cells in culture [11]. HEK PS DKO stably expressing GLT-1 GFP were plated at 0.5 × 10^5^ cells per well in 24-well tissue culture plates. Glutamate uptake experiments were performed 2 days after transfections. Cells are washed twice with sodium (Na) (140 mM) or choline (Ch) (140 mM) buffer, both containing 2.5 mM KCl, mM CaCl_2_, 1.2 mM MgCl_2_, 1.2 mM K_2_HPO_4_ and 10 mM glucose, buffered to pH 7.4 with sodium phosphate. l-[^3^H]-glutamic acid (catalog #NET490250UC, PerkinElmer; specific activity 50.8 Ci/mmol) was added at a final total (radioactive plus non-radioactive) glutamate concentration of 50 μM. To verify the specificity of glutamate uptake by GLT-1, cells were treated with an inhibitor of GLT-1 (DHK, 1 mM) or (WAY213613, 10 μM) and uptake compared with vehicle control. Cells were incubated at 37°C for 5 min; the uptake was terminated by washing cells twice in ice-cold choline buffer containing 1% BSA, and cells were solubilized in 0.1 mM NaOH for 30 minutes, 0.25 ml/well and lysate then transferred to liquid scintillation vials. Total protein content in lysates was determined by Pierce BCA protein assay (ThermoFisher). Radioactivity was assayed by liquid scintillation counting. In all experiments sodium-dependent uptake was determined by subtracting the values obtained in choline buffer from the values obtained in sodium buffer.

### Statistical Analysis

Statistical analysis was performed using GraphPad Prism 5 software (La Jolla, CA). ANOVA with Bonferroni’s post-hoc correction was used unless otherwise stated in the results. Values were considered significant at *p* < 0.05.

## Results

### Sporadic AD brains have reduced interaction between PS1 and GLT-1

**Figure 1:**
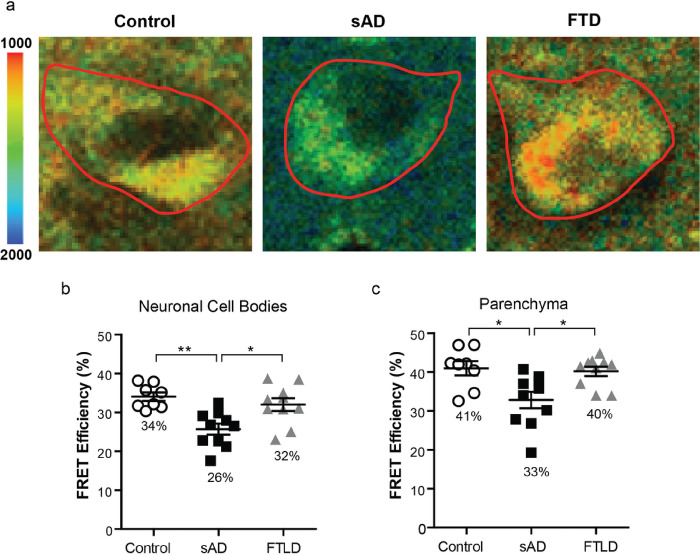
PS1/GLT-1 interaction changes in sporadic AD (a) Color-coded FLIM images of representative neurons of sAD, control and FTLD brain samples. Donor fluorophore lifetimes in neuronal cell bodies (outlined) and parenchyma (GLT-1 positive synaptic terminals and astrocytes) represent different proximity between fluorophore-tagged PS1 and GLT-1 and thus distance between PS1 and GLT-1 proteins. Colorimetric scale shows fluorescence lifetime in picoseconds (ps). FLIM analysis of the PS1/GLT-1 interaction (%E_FRET_) in frontal cortex neurons (b) and frontal cortex parenchyma (c) in human brain. Average %E_FRET_ in sporadic AD (n=10, ~30 neurons per case), non-demented control (n=8, ~30 neurons per case), and FTLD (n=10, ~30 neurons per case) human brain tissue. Bar shows mean±SEM, *=p<0.05, **=p<0.01. Kruskal-Wallis ANOVAs with Dunn’s multiple comparison test.

To test whether the PS1/GLT-1 interaction changes due to sporadic AD (sAD), the PS1/GLT-1 interaction was examined using FLIM in intact brain tissue of control, sAD and frontotemporal lobar degeneration (FTLD) patients. The brain sections were immunostained with anti-GLT-1 (Millipore, 1:250 ) and anti-PS1 (Sigma-Aldrich, 1:250) antibodies. The randomly selected neuronal cell bodies or “neuron-free” parenchyma containing neurites and astrocytes in the frontal cortex and hippocampus sections were outlined as the regions of interest (ROIs) in PS1/GLT-1 immunostained brain sections. The donor fluorophore lifetimes were measured as an indicator of proximity between the GLT-1 and PS1. The sAD tissue showed significantly less interaction between PS1 and GLT-1, measured by a decrease in FRET efficiency, compared to control or FTLD brains in both neuronal cell body-enriched and parenchyma ROIs (Fig. 1; Kruskal-Wallis ANOVA with Dunn’s multiple comparison test). Although the PS1/GLT-1 interaction was different, the pattern of GLT-1 and PS1 immunoreactivity seemed comparable between the control brains and brains of sAD patients despite severe neurodegeneration in the latter (Fig. S1a). The specificity of GLT-1 immunoreactivity in neuronal cell bodies in human brain was verified using additional anti-GLT-1 antibody, raised against N-terminus (nGLT-1) [11]. The nGLT-1 antibody recognizes both neuronal and astrocytic GLT-1; and was previously tested on brain sections from GLT-1 KO mice [61]. The strongest GLT-1 immunoreactivity derived from astrocytes and neuronal axons within the brain parenchyma, however occasional neuronal cell bodies also showed GLT-1 positivity (Fig. S1b). There was no significant effect of age, post-mortem interval (PMI), Braak stage or sex on the interaction in neuronal cell body-enriched or parenchyma ROIs (Fig. S2; Kruskal-Wallis ANOVA with Dunn’s multiple comparison test).

To further prove the PS1 and GLT-1 interaction occurs in the human brain, coimmunoprecipitation of the PS1/GLT-1 complexes from human brain lysates was carried out (Fig. S3a). The interaction was detected in Control, sAD and FTLD brains. Unlike with the FLIM assay, measurable difference in the amount of PS1 coimmunoprecipitated with GLT-1 was not detected (Fig. S3b). This divergence could possibly be due to the higher sensitivity of the cell-by-cell/ROI based FLIM technique, since the latter approach monitors relative proximity between proteins in their native environment without the need of lysis buffer and detergents, and can better capture more transient interactions.

#### PS1 conformational state modulates interaction with GLT-1.

**Figure 2:**
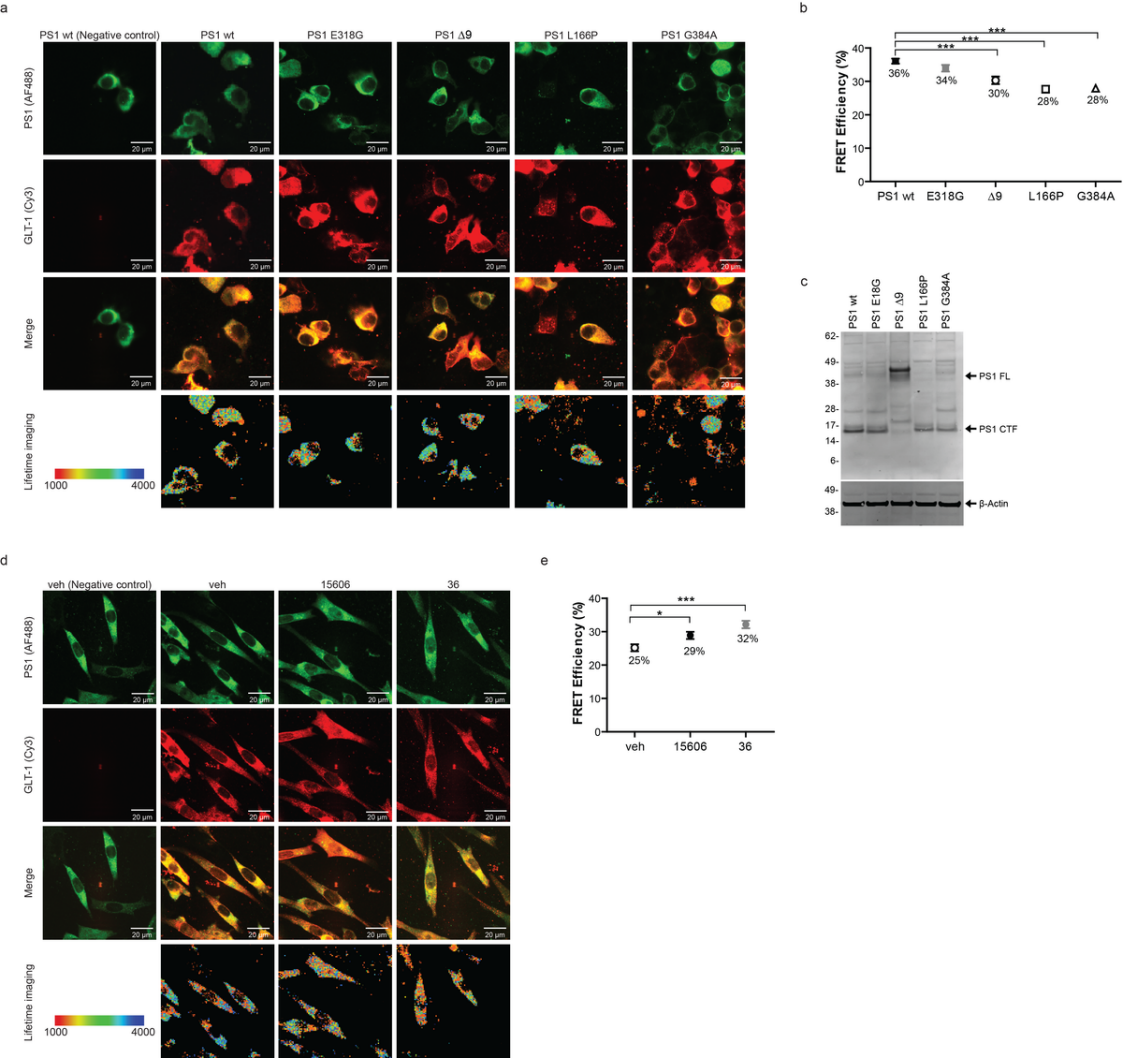
PS1 conformation impacts the PS1/GLT-1 interaction. (a,b) Pathogenic fAD PS1 mutations decrease the PS1/GLT-1 interaction. HEK PS DKO cells transiently co-transfected with GLT-1 and wildtype PS1 or PS1 with familial AD mutations. (a) Confocal images of HEK PS DKO cells co-transfected with GLT-1 and PS1 and immunostained with anti-PS1 and anti-GLT-1 antibodies (top three panels). Bottom panel shows color-coded FLIM images of the donor fluorophore lifetimes in the cell. Orange-red pixels, corresponding to the shorter fluorescence lifetimes, are indicative of the closest proximity between the fluorophore-labeled PS1 and GLT-1. The colorimetric scale shows AF488 fluorescence lifetime in picoseconds. (b) FLIM analysis of the PS1/GLT-1 proximity (%EFRET) in cells expressing PS1 wildtype, non-pathogenic E318G, and fAD Δ9, L166P or G384A mutants with GLT-1 (3 independent replications, n=108–112 cells per group). Error bars show ±SEM, ***=p<0.001. One-way ANOVA with Bonferroni’s post-hoc correction. (c) Western blot representing the level of PS1 wt and PS1 fAD in presence of GLT-1. HEK PS DKO have been co-transfected with GLT-1 and PS1 wt or non-pathogenic E318G, or fAD Δ9, L166P or G384A mutants. (d,e) FLIM analysis of the PS1/GLT-1 proximity in CHO cells treated with PS1 conformation modifying gamma-secretase modulators (GSMs) reveals stronger FRET efficiency (%EFRET). (d) Top panels show cells transiently transfected with wildtype PS1 and GLT-1 DNA, and immunostained with anti-PS1 and anti-GLT-1 antibodies. Bottom panel shows color-coded FLIM images of the donor fluorophore lifetimes in the cell. Orange-red pixels, corresponding to the shorter fluorescence lifetimes, are indicative of the closest proximity between the fluorophore-labeled PS1 and GLT-1. The colorimetric scale shows AF488 fluorescence lifetime in picoseconds. (d) FLIM analysis (%EFRET) of GSM15606, GSM36, or vehicle control treated cells (4 independent replications, n=108–112 cells per group). Error bars show ±SEM, *=p<0.05, ***=p<0.001. One-way ANOVA with Bonferroni’s post-hoc correction.

PS1 has been known to adopt the pathogenic closed conformation in sAD, but not in FTLD, and as a result of fAD PS1 mutations [5, 64, 69]. Alternatively, treatment with NSAIDs or gamma-secretase modulators (GSMs) lead to a more relaxed, open PS1 conformation [39, 64]. Thus, we wanted to first examine if PS1 pathogenic mutations disrupts the interaction between PS1 and GLT-1. HEK PS DKO cells were transiently co-transfected with GLT-1 and either wildtype or mutant PS1 (Fig. 2c), and the PS1/GLT-1 proximity was evaluated in intact cells by FLIM. Of note, the FLIM assay is not concentration-dependent [32, 34], and FRET efficiency (proximity between PS1 and GLT-1) is not impacted by PS1 wt or fAD expression level. HEK PS DKO co-expressing GLT-1 with PS1 wt or fAD PS1 Δ9, L166P and G384A mutants were stained with Cy3- and AF488-fluorophore labeled antibodies to detect the GLT-1 and PS1, respectively (Fig. 2a top two panels). High-resolution FLIM generated a color-coded image of the AF488 donor fluorophore lifetimes within each cell, reflecting proximity between the PS1 and GLT-1 (Fig. 2a bottom panel). We found that all fAD PS1 mutations tested decreased the PS1/GLT-1 interaction, measured by a decrease in FRET efficiency (Fig. 2b; one-way ANOVA with Bonferroni’s *post-hoc* correction). Interestingly, in cells expressing non-pathogenic mutation E318G, the interaction between PS1 and GLT-1 did not differ from that in cells expressing wildtype PS1.

Next, we tested if altering PS1’s conformation to be more open and non-pathogenic would impact the protein binding to GLT-1. We used GSM15606 and GSM36 to induce more “relaxed” PS1 conformations in CHO cells transiently co-transfected with GLT-1 stained with Cy3 and wildtype PS1 stained with AF488 (Fig. 2d). GSM treatment with either GSM15606 or GSM36 increased the PS1/GLT-1 interaction, measured by an increase in FRET efficiency, compared to vehicle treated cells (Fig. 2e; one-way ANOVA with Bonferroni’s *post-hoc* correction). These results indicate that PS1 conformation and possibly its structural arrangement may be important for the binding of PS1 to GLT-1.

### PS1 increases GLT-1 cell surface expression and multimerization

**Figure 3:**
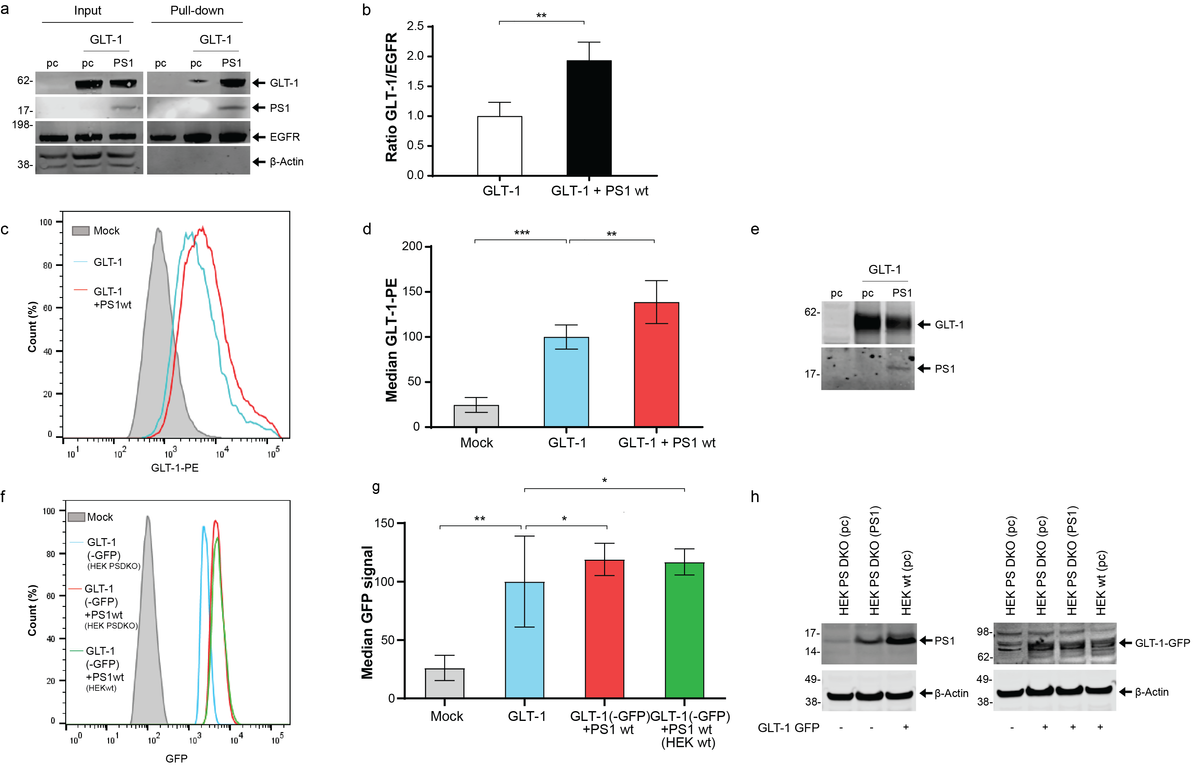
PS1 increases GLT-1 cell surface expression (a) Representative western blots showing GLT-1 and PS1 total expression (input) and cell surface expression (Pull-down) in HEK PS DKO cells co-transfected with GLT-1 and either pcDNA3.1-PS1 or pcDNA3.1-GFP (pc). Left lane (pc) shows HEK PS DKO cells transfected with pc alone. The cells were biotinylated, lysed, and streptavidin was used for pull-down. Membranes were probed for GLT-1, PS1 CTF, EGFR and β-actin. (b) The presence of PS1 increases GLT-1 cell surface expression compared to GLT-1 in pcDNA3.1-GFP (pc) transfected cells. Biotinylation band intensity was equalized to cell surface EGFR, and normalized to GLT-1+pc in the input. Four independent replications per group, bars show mean±SEM. One-way ANOVA with Bonferroni’s post-hoc correction. **p<0.01. (c) Representative Flow cytometry analysis of cell surface GLT-1 in CHO cell lines expressing GLT-1 and pcDNA3.1 (in blue) or co-expressing GLT-1 and PS1 (in red). Mock (in grey) represents Rabbit IgG Isotype control on pcDNA3.1 transfected cells. Cells were fixed with 2% PFA without permeabilization and incubated with anti-GLT-1 antibody and PE labelled secondary-antibodies. The labelled cells were gated in regard to GLT-1-PE signal. (d) Analysis of the median cell surface GLT1-PE immunofluorescence signal normalized to total GLT-1. N=3 independents experiments. bars show mean ± SEM. One-way ANOVA with Bonferroni’s post-hoc correction. **p<0.01, ***p<0.001. (e) Representative western blots showing GLT-1 and PS1 expression in CHO cells used for Flow cytometry. (f) Representative Flow cytometry analysis of GLT-1 cell surface in HEK cells. HEK PS DKO stably expressing GLT-1 GFP were transfected with pcDNA3.1 (in blue) or PS1wt (in red). Naïve HEKwt cells stably expressing GLT-1 GFP and PS1 at endogenous level are shown (in green). Mock (in grey) represents pcDNA3.1 empty vector transduced cells. Cells were analyzed alive and gated in regard to GLT-1-GFP signal. (g) Analysis of the median cell surface GFP signal was normalized to total GLT-1-GFP.N=3 independents experiments. bars show mean ± SEM. One-way ANOVA with Bonferroni’s post-hoc correction. *p<0.05, **p<0.01. (h) Representative western blots showing GLT-1-GFP and PS1 expression in HEK PS DKO and HEK wt cells used for Flow cytometry. The blot on the left compares the expression level of PS1 transiently transfected in HEK PS DKO vs the level of endogenous PS1 in HEK wt. The right blot compares the expression level of stably transduced GLT-1 GFP (+) in HEK PS DKO, with or without PS1 vs. HEK wt cell line.

To determine if PS1 and its binding to GLT-1 may impact GLT-1 expression at the outer surface of the plasma membrane, where functional GLT-1 resides [1] we employed two complementary approaches. HEK PS DKO cells were transiently co-transfected with GLT-1 and PS1 or GLT-1 and pcDNA3.1-GFP vector (pc). Transfection of the HEK PS DKO cells with pcDNA3.1-GFP alone was used as a negative control. Intact cells were biotinylated to label the cell surface proteins, lysed, and biotin-tagged proteins were pulled down with Streptavidin. Western blotting with GLT-1 antibody detection was used to measure the effect of the presence of PS1 on GLT-1 levels at the cell surface. The amount of cell surface (biotinylated) GLT-1 protein was normalized to the level of cell surface EGFR in the same sample. We found increased GLT-1 cell surface expression in the presence of PS1 compared to that in pc control (Fig. 3a–b).

We have also used an alternative approach, flow cytometry, to examine the effect of PS1 on cell surface GLT-1 in a different cell line, CHO cells transiently co-transfected with GLT-1 and pcDNA3.1 or with GLT-1 and PS1. The cells were fixed and stained with GLT-1 antibody without permeabilization, followed by PE-conjugated secondary antibody. Cells transfected with pcDNA3.1 empty vector (Mock) were labeled with IgG from rabbit as isotype negative control. The relative fluorescence intensity at the membrane surface of CHO expressing GLT-1-PS1 was increased in comparison to CHO cells expressing GLT-1 alone (Fig. 3c–d). The western blot analysis shows comparable levels of total GLT-1 expression in CHO cells with and without transfected PS1 (Fig. 3e).

Since CHO cell’s endogenous PS1 may have skewed the findings, we have confirmed these results using HEK PS DKO cells lacking both, PS1 and PS2, and stably expressing GLT-1 GFP. Live HEK PS DKO GLT-1 GFP cells were transfected with either pcDNA3.1 or PS1 (blue and red graphs in Fig. 3f,g). Furthermore, to verify that endogenously expressed PS1 would also affect the cell surface levels of GLT-1, we compared HEK wt cells stably expressing GLT-1 GFP (green in Fig. 3f,g) to HEK PS DKO GLT-1 GFP cells (blue in Fig. 3f.g).

All flow cytometry experiments have shown similar results: more GLT-1 (GFP or PE labeled) appear at the cell surface in the presence of either endogenous or transiently expressed PS1, as compared to cells expressing GLT-1 alone. We have verified by Western blot that stable GLT-1-GFP is expressed at comparable levels in HEK wt and in HEK PS DKO cell lines (Fig. 3h, right). The level of PS1 endogenously expressed in HEK wt or transiently transfected into HEK PS DKO is shown in Fig. 3h, left. Based on both biotinylation and flow cytometry assays, we can conclude that the presence of PS1 facilitates GLT-1 cell surface delivery.

**Figure 4:**
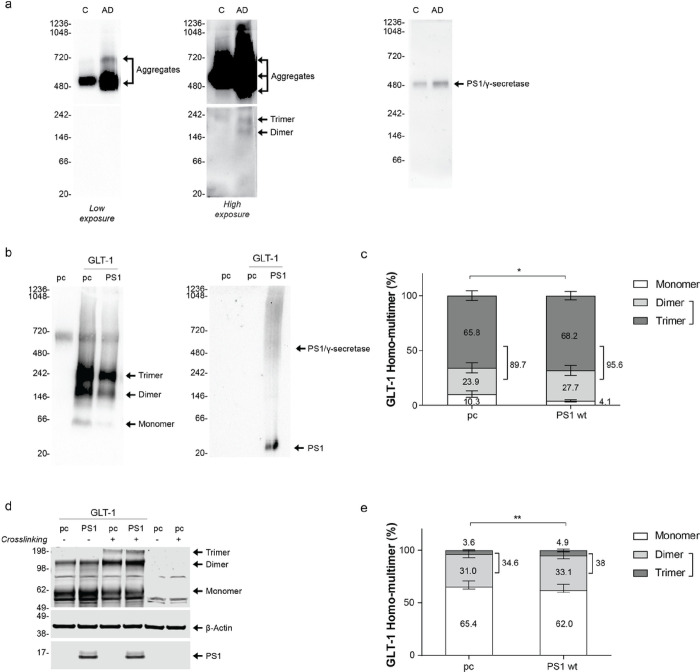
PS1 induces GLT-1 multimerization (a) Representative immuno-blot after Blue Native Polyacrylamide Gel Electrophoresis (BN/PAGE) showing GLT-1 expression profile in Control vs AD human brain lysates (n=4). First lane corresponds to control (C) and second lane Alzheimer’s disease case (AD). The first two blots are probed with GLT-1 antibodies (Abcam); Intense bands are detected above 480 kDa and corresponding to GLT-1 aggregates. On high exposure blot, above 146 kDa and 242 kDa bands correspond to dimer and trimer respectively. Third blot is probed for PS1 (BioLegend) and shows PS1/γ-secretase complex. (b) Representative immuno-blot after BN/PAGE showing GLT-1 expression profile co-expressed with pcDNA3.1 (pc) or PS1. First lane corresponds to HEK PS DKO cells transfected with “pc” only. HEK PS DKO cells were treated with 5% digitonin prior BN/PAGE and Western blotting for GLT-1 detection. GLT-1 antibodies indicate 70 kDa, ~146 kDa and 242 kDa bands corresponding to monomer, dimer and trimer respectively. Second blot is probed for PS1 (BioLegend) and shows PS1/γ-secretase complex and PS1. (c) Quantification of relative intensity of GLT-1 monomer, dimer and trimer in absence (pc) or presence of PS1. The level of multimerization profile is measured in comparison of monomer vs dimer+trimer. Bars show mean ±SEM. N=3 independent experiments in triplicates. *p<0.0231. Two-way ANOVA with Sidak’s multiple comparisons test. (d) Representative Western blots of HEK PS DKO cells, transiently transfected with GLT-1 and empty DNA vector (pc), or wildtype PS1. Cross-linking agent, DSS (disuccinimidyl suberate) (+), was used at 50 μM to stabilize GLT-1 multimers. (−) corresponds to cells treated with DMSO only. Membranes were probed for GLT-1, PS1 and β-actin as a loading control. GLT-1 antibody revealed 55 kDa, 120 kDa and 190 kDa bands corresponding to monomer, dimer and trimer respectively.(e) Quantification of the relative intensity of GLT-1 monomer, dimer and trimer bands crosslinked (Image Studio Lite). N=6 independent experiments, bars show mean ± SEM **p<0.01. Two-way ANOVA with Sidak’s multiple comparisons test.

GLT-1 multimerization occurs during the transporter maturation and is required for the transporter functional activity [24, 28, 76]. GLT-1 multimerization profile has been verified in human brain tissue. Human brain lysates, Control vs AD cases, were ran in non-denaturing condition using Native Blue Page (NB) assay (Fig. 4a). Interestingly, we observed that AD cases presented more PS1/ γ-secretase (Fig. 4a, right), and increase in GLT-1 dimer (~ 160 kDa) and trimer (~ 242 kDa) (Fig. 4a center, bottom image) but also more GLT-1 aggregates that are present above 480 kDa (Fig. 4a, left and center images). To determine whether PS1 could alter homomultimerization of GLT-1 we have studied GLT-1 profile in HEK PS DKO cells after transient transfection of pcDNA3.1 GFP (pc) or co-transfection of GLT-1 with either pc or wildtype PS1 in non-denaturing condition. After probing with GLT-1 antibody we observed bands at around 70 kDa, 160 kDa and 242 kDa corresponding to monomer, dimer and trimer, respectively (Fig. 4b). We have quantified the signal of monomer/dimer/trimer (total GLT-1 expression) and determined the relative intensity of each band as a percentage (Fig. 4c). In the absence of PS1, the amount of GLT-1 multimers, the functional form of GLT-1, represented 89.7% of the total GLT-1 expression. When GLT-1 is co-expressed with PS1, the GLT-1 multimers increased to 95.6% of the total GLT-1 expression. The increase of the GLT-1 homomultimers, especially functionally active trimers, relative to the level of GLT-1 monomers in the presence of PS1 was statically significant (Fig. 4c; Two-way ANOVA with Sidak’s multiple comparisons test). To verify further if GLT-1 multimerization profile is affected by PS1 we used a different approach – Western blotting in denaturing conditions after treating living cells with disuccinimidyl suberate (DSS) (+) to stabilize GLT-1 homomultimers (Fig. 4d). Cells were also treated with DMSO only (−) to confirm the multimers observed came from the DSS stabilization. Cells were lysed in 1% NP-40 + 0.1% Triton-X and resolved by SDS-PAGE. Trimers were only observed in the presence of DSS treatment. The GLT-1 monomer/dimer/trimer bands in DSS treated cells with and without PS1 were quantified after immunoblotting with GLT-1 antibody. We found that when GLT-1 is co-expressed with PS1, the percentage of GLT-1 monomer level slightly decreased, while the dimer and trimer sum correspondingly increased. The increase in the fraction of GLT-1 homomultimers (dimer + trimer) in the presence of PS1 was statically significant as compared to that in cells without PS1 (Fig. 4e; Two-way ANOVA with Sidak’s multiple comparisons test).

### PS1 does not impact GLT-1 stability

**Figure 5:**
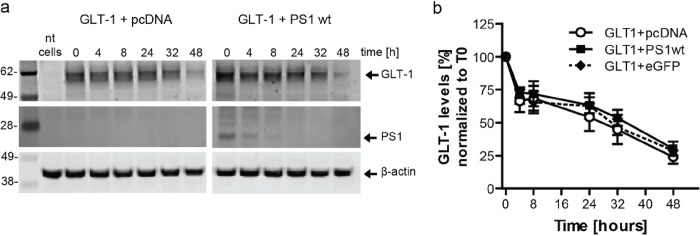
PS1 does not impact GLT-1 protein stability. (a) Representative western blots of CHO cells transiently transfected with GLT-1 and wildtype PS1 or an empty vector (pcDNA) treated with cycloheximide (CHX) and lysed at various time points to determine relative GLT-1 protein stability. eGFP was also transfected with GLT-1 as another control (irrelevant protein) (representative WB not shown). (b) PS1 did not significantly impact GLT-1’s protein stability, as measured by the decrease of GLT-1 protein over time after treatment with CHX. 5 independent replications per group, bars show mean±SEM. Two-way ANOVA followed by Dunnett’s multiple comparisons.

To determine if the effect of PS1 on increased GLT-1 cell surface expression was due to an increase in GLT-1 protein stability, a cycloheximide chase assay was performed with CHO cells transiently co-transfected with GLT-1 and either an empty pcDNA vector (pc) or wildtype PS1. The decrease in GLT-1 protein was measured over the course of two days at various timepoints using Western blotting with GLT-1 antibody detection. PS1 did not significantly impact GLT-1’s protein stability (Fig. 5a–b).

### PS1 does not modulate glutamate uptake mediated by GLT-1 after 5 min glutamate treatment

**Figure 6:**
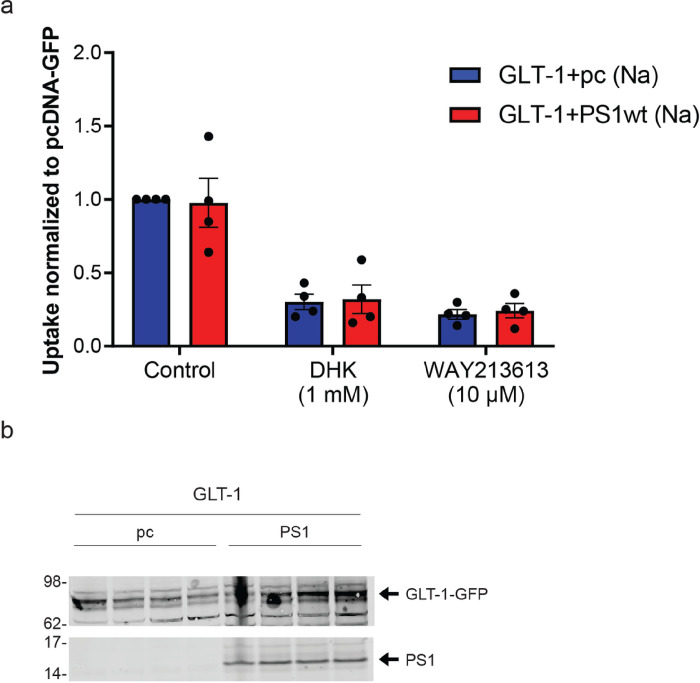
PS1 does not alter GLT-1 mediated glutamate uptake in HEK cells after 5 minutes glutamate treatment. ^3^H-L-glutamate uptake was assayed in HEK PS1 DKO cells stably expressing GLT-1. Cells were transfected with PS1 or control vector (see methods). Two days after transfection, experiment was performed in which cells were exposed to ^3^H-L-glutamate in a total glutamate concentration of 50 μM for 5 minutes at 37°C. In each experiment, there were 3 groups of cells, two in which the GLT-1 specific inhibitors DHK (1 mM) or WAY213613 (10 uM) were present, and the third in which vehicle only was present. Four separate experiments were performed, each representing a separate passage and plating of the HEK cells, and the results shown are data pooled from those 4 experiments. Results are all normalized to the control (vehicle only) value from the PS1 negative (pc DNA-GFP) condition. Transport activity was inhibited by specific inhibitors of GLT-1. There was no difference in glutamate uptake between genotypes. Error bars show SEM (b) Western blot of cell lysate used for GLT-1 glutamate uptake. Membrane was probe with GLT-1 (ABCAM) antibody and PS1 antibody (CST).

To determine if PS1 can modify GLT-1 glutamate uptake, we compared uptake of ^3^H-L-glutamate into HEK PS DKO cells stably expressing GLT-1 GFP (HEK PS DKO GLT-1) and transfected either with PS1 wt or pcDNA3.1 (pc). Glutamate uptake by the plasma membrane glutamate transporters is highly sodium dependent [75], and we determined the sodium dependent component of glutamate uptake in these experiments by measuring uptake in a sodium containing uptake buffer or in an uptake buffer in which sodium chloride was replaced by choline chloride. Total radioactive and non-radioactive glutamate was present at a concentration of 50 μM. The effect of GLT-1 inhibitors DHK (1 mM) or WAY213613 (10 μM) was assessed in cells assayed in parallel with cells not treated with inhibitors to verify that uptake was mediated by GLT-1 (Fig. 6). In four experiments, we found no consistent effect of PS1 wt expression on the uptake of glutamate measured at 5-minute time point.

### GLT-1 interacts with PS2

PS1 and PS2 are highly homologous polytopic membrane proteins. Since we discovered that GLT-1 interacts with PS1, we next explored if GLT-1 may also bind to PS2. First, to ensure PS1 does not interfere with the coimmunoprecipitation, we used HEK PS DKO transiently co-transfected with GLT-1 and either PS2 wt or pcDNA 3.1. IgG was used as a negative control for immunoprecipitation. Western blot analysis of HEK PS DKO using the anti-PS2 C-terminal antibody for detection demonstrated the presence of a PS2-CTF band in the GLT-1 immunoprecipitated fraction (Fig. S4a), suggesting that the binding between the GLT-1 and PS2 may also occur.

To verify the GLT-1/PS2 interaction at endogenous level, we used primary neurons and mouse brain lysates. Endogenous GLT-1/PS2 complexes were detected by co-immunoprecipitation in neurons *in vitro* and in mouse brain tissue (Fig. S4b and c, respectively). In addition, we confirmed the GLT-1/PS2 interaction by an alternative approach, FLIM analysis in intact neurons (Fig. S4d). The median lifetime of AF488 (FRET donor) in neurons with AF488 labeled PS2 only is 2528 +/− 290.1 ps, while AF488/PS2 lifetime in cells co-immunostained with Cy3/GLT-1 shortened to 2247 +/− 172.8 ps. Shortening of the donor fluorophore lifetime indicates FRET signal, and thus close proximity between AF488/PS2 and Cy3/GLT-1.

## Discussion

We recently found that PS1 and GLT-1 interact in the mouse brain, and this interaction occurs in both astrocytes and neurons cultured *in vitro* [77]. In the current study, we first established that PS1/GLT-1 interaction also occurs in the human brain. Interestingly, this interaction diminishes in the brain of sporadic AD patients compared to non-demented controls, as measured by FRET-based approach in intact tissue. Further, we compared PS1/GLT-1 interaction in sAD to FTLD, another dementia/neurodegeneration condition. FTLD may or may not have tau inclusions (NTF in Table 1), but unlike sAD, does not have amyloid pathology. Intriguingly, PS1/GLT-1 interaction was not significantly altered in FTLD brain and was comparable to that of controls. This indicates the specificity of diminished PS1/GLT-1 interaction to sAD and to amyloid pathology, by extension.

PS1 adopts pathogenic “closed” conformation due to fAD PS1 mutations and in sAD, but not in FTLD brains [5, 64, 69]. Thus, we investigated the hypothesis that PS1 conformation disrupts PS1/GLT-1 interaction in sAD brain. For this, we used two complementary approaches known to affect PS1 conformation: fAD PS1 mutations, which induce a “closed” pathogenic conformation, and γ-secretase modulators, GSMs, which induce an “open” conformation [55, 64]. Our findings indicate that PS1 conformation has a direct effect on PS1/GLT-1 interaction, with fAD mutations reducing the interaction, and GSM treatment increasing the interaction. Of note, non-pathogenic PS1 E318G mutation, which does not change PS1 conformation [5], did not significantly alter PS1/GLT-1 interaction. This suggests PS1/GLT-1 interaction is dynamically regulated by PS1 conformation, and changes in PS1 conformation due to aging, oxidative stress [2], or fAD mutations [5, 69] disrupt the structural arrangement of PS1 binding to GLT-1. Importantly, pathogenic changes in PS1, that result in a decrease in the PS1/GLT-1 interaction in AD, support the idea that this interaction may be beneficial. Plausibly, gradual and continued reduction in PS1/GLT-1 interaction occurring in AD brain, disrupts the localization or maturation of this glutamate transporter, as suggested by our *in vitro* data. This would help explain the strong association between fAD PS1 mutations and epileptiform activity [35] and more generally the hyperactivity of neural circuits observed in both fAD and sAD [7, 47, 54].

The age, sex, Braak stage of the sAD cases, or PMI of the samples studied did not significantly affect PS1/GLT-1 interaction as measured by the FLIM assay. Regarding the ApoE genotype of the subjects, six of the eight AD cases used herein, with known ApoE, present at least one ApoE4 allele, whereas only one out of five FTLD cases was ApoE4 positive and all three C with known ApoE status were ApoE 3/3 (Supplementary Table 1). Thus, we cannot exclude whether ApoE can modulate PS1/GLT-1 interaction in direct or indirect manner by, for example, inducing a PS1 conformational change. However, since direct or indirect effect of ApoE on PS1 conformation has never been reported in the literature, and since the ApoE status is unknown for most of the FTLD and C cases – we currently cannot make such conclusion.

The reported data on the levels of GLT-1 protein in human brain are somewhat controversial, even for those studies that investigated the same brain regions [72]. Previous studies found no change in GLT-1 protein levels in the human frontal cortex [18] as well as the medial prefrontal cortex in an AD mouse model [31]. Whereas other research has described significant decrease in GLT-1 expression in the frontal cortex of AD patients [38] as well as a decrease in GLT-1 gene and protein expression in the hippocampus of sAD patients [26]. It was also reported, that GLT-1 aggregates are elevated in AD brains [71], and that oxidation damage to GLT-1, including oxidation damage as a result of exposure to Aβ [21, 29, 36], results in high-molecular weight GLT-1 oligomers [63]. Our findings agree with the findings of Woltjer et al., (2010), and show significantly higher levels of GLT-1 aggregates in sAD brain, as detected by Native Blue PAGE assay. Detergent-soluble GLT-1 levels were reduced in AD compared to control brains, suggesting that GLT-1 may be a protein that exhibits altered solubility in AD [71]. This change in GLT-1 solubility and expression may explain the conflicting results surrounding GLT-1 levels in the AD brain.

We found that control brains, besides aggregates, contained mainly GLT-1 trimers (functional form), whereas AD brains also had significant amount of less mature GLT-1 dimers. In addition, we observed that PS1/γ-secretase complexes increase in AD human brain tissue compared to control cases, while transcriptomic analysis shows no impact on mRNA level [65]. This observation suggests, along with pathogenic “closed” PS1 conformation that prevents efficient binding to GLT-1, PS1 may misfold/accumulate in AD brain and cannot provide proper chaperoning activity leading to GLT-1’ mislocalization and increased aggregation.

An important consideration is whether the interaction is relevant when it happens in astrocytes, neurons, or both. Although high levels of mRNA is detected in neurons [6, 53], GLT-1 protein is expressed more abundantly on astrocytes than on neurons and historically has been treated as primarily an astrocytic transporter [10, 37]. However, more recently GLT-1 has been found to have an impactful role in neurons that is disproportionate to its expression [42, 50, 56]. *In vivo*, neuronal GLT-1 primarily localizes to axons and synapses [17]; however, low levels of GLT-1 protein can be detected in cell bodies of some, presumably stressed neurons in the brain [51, 62]. We previously detected PS1/GLT-1 interaction in mouse primary astrocytes and primary neurons [77], and the current study detected this interaction in human tissue in both the parenchyma (GLT-1-positive astrocytes and axons) and in occasional neuronal cell bodies. Therefore, considering these results, we can conclude that this interaction occurs in both neurons and astrocytes but not if this interaction differs significantly between the two cell types pertinent to regulatory and functional consequences.

GLT-1 activity is regulated at multiple levels, including total protein expression, trafficking to the cell surface, and multimer formation [1]. To determine if PS1/GLT-1 interaction may influence GLT-1 localization at the cell surface, and therefore could indicate changes in GLT-1 maturation and function, we measured the effect of PS1 on GLT-1 cell surface expression, homomultimerization, stability, and glutamate uptake. At the cellular level, we found that the presence of PS1 increased GLT-1 multimer formation and GLT-1 cell surface expression. Our findings indicate that these changes were not due to an alteration in GLT-1 stability, as the presence of PS1 did not noticeably impact GLT-1 protein stability.

An important question that arises from this finding is whether changes in PS1/GLT-1 interaction result in a direct measurable modification of GLT-1 function. The challenge with experiments aimed to address this question is that synaptic dysfunction due to glutamate transport impairments in AD brain occurs chronically, over many years, possibly decades, whereas the current models of glutamate uptake report more acute changes. Our *in vitro* data suggest that PS1 directly interacting with GLT-1 may act as a chaperone for GLT-1, promoting or stabilizing its homo-multimerization and cell surface delivery, and potentially modulating GLT-1-mediated glutamate uptake, over time. We did not observe significant difference in the glutamate uptake between HEK PS DKO cells lacking PS1/PS2 and HEK PS DKO cells transiently transfected with human PS1, as assayed by measuring ^3^H-L-glutamate uptake after 5-minute treatment with glutamate. However, these results do not rule out the possibility of a more long-term effect of the disrupted PS1/GLT1 interaction. Given that PS1/GLT-1 interaction is reduced in sAD brain leading to GLT-1 aggregation, it is possible,that it also impairs GLT1 cell surface trafficking in AD. This would have functional implication on glutamate uptake, and thus play a key role in excitotoxicity and neurodegeneration. Indeed, abnormal glutamate transport and hyperactivity have been reported as early events in AD, with high incidence of epileptic seizures in presymptomatic fAD mutations carriers [7, 40, 54, 67]. Furthermore, this study may provide a mechanistic insight into the previous findings that AD patients with PS1 mutations have significantly higher epileptiform activity [35, 47, 67]. Relevantly, the presence of Aβ disrupts glutamate transmission at the synapse and reduces local GLT-1 expression [13, 25, 58, 71]. Aβ-containing AD brain extracts can induce neuronal hyperactivity [78]. A recent transcriptomic study using human AD tissue has shown that hyperactivity is a prelude to a subsequent excitatory neuronal loss in their Aβ positive samples, and this cell state is specific to early AD pathology [19]. In AD mouse models, GLT-1 upregulation rescued cognitive decline [79] and GLT-1 loss increased cognitive decline [45], suggesting treating GLT-1 dysregulation might alleviate cognitive deficits observed in AD. In this scenario, a pathogenic conformational change in PS1 leads it to act as a faulty chaperone, failing to properly bind to and aid GLT-1 to reach the cell surface and/or to induce GLT-1 multimerization, which would compromise GLT-1 function.

Moreover, there could be other consequences of disrupted PS1/GLT1 interaction in sAD brain. For example, the synaptic protein PICK1 interacts with the isoform GLT-1b [3]. Subsequently, it was found that co-expression of PICK1 with GLT-1b in oocytes induced a substantial leak current mediated by GLT-1b [59]. GLT-1 expressed in astrocytes [20] and neurons [42, 43] has important metabolic functions, and metabolic consequences of the PS1/GLT1 interaction might not be discernible unless specifically looked for.

In conclusion, the current study establishes PS1/GLT-1 interaction occurs in the human brain and is disrupted in sAD, but not in FTLD. Additionally, the interaction may be important for GLT-1 multimerization and cell surface expression, and this interaction is dynamically regulated by PS1 conformation. Therefore, disruption of PS1/GLT-1 interaction due to PS1 conformational changes, either in fAD or sAD, could be a cause of impaired GLT-1 function and ultimately glutamate dyshomeostasis, providing a mechanistic link between glutamate transporter dysfunction and amyloid pathology. Thus, targeting PS1/GLT-1 interaction can be a potential new strategy for therapeutic intervention in AD.
